# Management of iron deficiency anemia and the role of intravenous iron supplementation in patients undergoing cancer chemotherapy: a real-world retrospective study in Japan

**DOI:** 10.1007/s10147-026-03057-4

**Published:** 2026-06-02

**Authors:** Miyako Kosugi, Ryo Takezawa, Shun Shiota, Shoichiro Inokuchi, Hirokazu Takaya, Masami Yamauchi

**Affiliations:** 1 Data Science Department, Zeria Pharmaceutical Co., Ltd., Tokyo, Japan; 2 Real World Evidence Division, Pharmaceutical Division, JMDC Inc., Tokyo, Japan; 3 Division of Clinical Oncology, Hiroshima Prefectural Hospital, Hiroshima, Japan

**Keywords:** Chemotherapy-induced anemia, Functional iron deficiency, Intravenous iron, Real-world data, Iron-deficiency anemia, Cancer-related anemia

## Abstract

**Background:**

Anemia is one of the common complications in patients with cancer, particularly among those undergoing systemic chemotherapy. Current guidelines for chemotherapy-induced anemia (CIA) recommend anemia evaluation and treatment, including assessing iron deficiency. However, actual pre- and post- chemotherapy clinical practices in Japan remain unclear. This retrospective observational study investigated real-world CIA management.

**Methods:**

In this descriptive study, we included patients with solid tumors who underwent antineoplastic therapy between January 2015 and March 2025. Baseline and follow-up hemoglobin (Hb) levels, transferrin saturation (TSAT), and serum ferritin levels were assessed. We also evaluated the anemia treatment status before and after chemotherapy, and changes in Hb levels following iron supplementation before chemotherapy.

**Results:**

In total, 75,603 patients were included. At baseline, 55.1% of patients had Hb levels below the lower limit of normal, and 11% presented with moderate-to-severe anemia (Hb < 10 g/dL). Iron parameters were measured in < 10% of patients. Among them, functional iron deficiency (TSAT < 50% with ferritin ≥ 30 and ≤ 500 ng/mL) was the predominant finding, increasing from 54.8% to 61.6% after chemotherapy initiation. Nevertheless, intravenous iron therapy was prescribed in < 3% of patients. An increase in Hb levels was observed in certain patients who prescribed intravenous iron 4–8 weeks prior to chemotherapy, including those with initially low Hb levels.

**Conclusion:**

This study demonstrated that anemia is highly prevalent in patients with cancer and worsens following chemotherapy. Additionally, iron parameters were rarely measured, and functional iron deficiency was frequently observed among the tested subgroup.

**Supplementary Information:**

The online version contains supplementary material available at 10.1007/s10147-026-03057-4.

## Introduction

Anemia is one of the common complications in patients with cancer, with its prevalence varying widely depending on the cancer type, stage, and the definition of anemia [1, 2]. Previous studies have reported that approximately 20–40% of untreated cancer patients and 50–90% of those receiving chemotherapy experience anemia [3, 4]. The causes of anemia in these patients are diverse and include blood loss, bone marrow infiltration, nutritional deficiencies (e.g., iron, folic acid, and vitamin B_12_) that impair hematopoiesis, chronic inflammation, chemotherapy-induced myelosuppression, and nephrotoxicity. Therefore, a careful investigation of the underlying conditions is required before initiating chemotherapy [5–7].

Iron deficiency is observed in 32–60% of patients with cancer, most of whom also meet the criteria for iron-deficiency anemia [8]. To assess anemia with iron deficiency, transferrin saturation (TSAT) and serum ferritin levels are generally recommended, in addition to hemoglobin (Hb) and red blood cell (RBC) indices [9–11]. TSAT represents the iron supply required to maintain normal RBC production and can be calculated using serum iron and total iron binding capacities (TIBC) or unsaturated iron binding capacities (UIBC) [12]. Ferritin reflects body iron stores but can be falsely elevated in patients with cancer due to the possibility of chronic inflammation [13].

Anemia that occurs during anticancer drug therapy is termed chemotherapy-induced anemia (CIA). Two major guidelines exist for CIA management, issued by the National Comprehensive Cancer Network (NCCN) [14] and European Society for Medical Oncology (ESMO) [15]. While the specific diagnostic thresholds and treatment strategies differ, both evaluate and treat patients based on a distinction between absolute iron deficiency (AID), indicating depletion of total body iron stores, and functional iron deficiency (FID), indicating sufficient iron stores but limited bioavailable iron for erythroblast production. In these guidelines, AID is defined as low TSAT and ferritin levels, with recommended treatment involving intravenous (IV) or oral iron supplementation. However, oral iron often slowly improves iron deficiency, especially in patients with cancer, due to impaired iron absorption from the gastrointestinal tract and insufficient iron release from stores [16–18]. Conversely, FID is defined by a low-to-moderate TSAT and normal ferritin levels, with recommended treatment involving IV iron with an erythropoietin-stimulating agent (ESA), or RBC transfusion. However, in Japan, ESAs are not approved in patients with CIA, making IV iron or RBC transfusions the sole treatment options available. Internationally, cancer studies including patients undergoing chemotherapy have shown that high-dose IV iron monotherapy can increase Hb levels, even in patients with low Hb levels (< 10 g/dL), and reduce the need for RBC transfusion [19–22]. However, no large-scale studies have reported the efficacy of iron therapy in patients with CIA in Japan. Furthermore, Japan lacks official guidelines for CIA treatment, and anemia management before and after chemotherapy initiation remains unclear.

Therefore, this study aimed to descriptively assess the current anemia evaluation and treatment practices using Japanese real-world data (RWD). We particularly focused on the dynamics of iron deficiency before and after chemotherapy initiation in patients with cancer and examined areas for improvement to achieve ideal anemia management.

## Patients and methods

### Data source

This study utilized a RWD database maintained by the Health Care and Education Information Initiative (HCEI; Kyoto, Japan), a joint research institute of JMDC Inc. (Tokyo, Japan) [23]. The HCEI database contains > 13 million electronic medical records (including admission and discharge, drugs, disease names, laboratory values, and basic patient information), Diagnosis Procedure Combination (DPC) data, and insurance claims data from > 90 medical institutions across Japan (including small clinics and large hospitals with > 1,000 beds). These records were classified according to the International Classification of Diseases 10th revision (ICD-10), the World Health Organization Anatomical Therapeutic Chemical (WHO ATC) classification system, and procedure/category codes. This study adhered to the principles of the Declaration of Helsinki and was approved by the Ethics Committee of MINS Research, a non-profit organization (Tokyo, Japan; approval number: MINS-IRB-240219).

### Study population

**Fig. 1:**
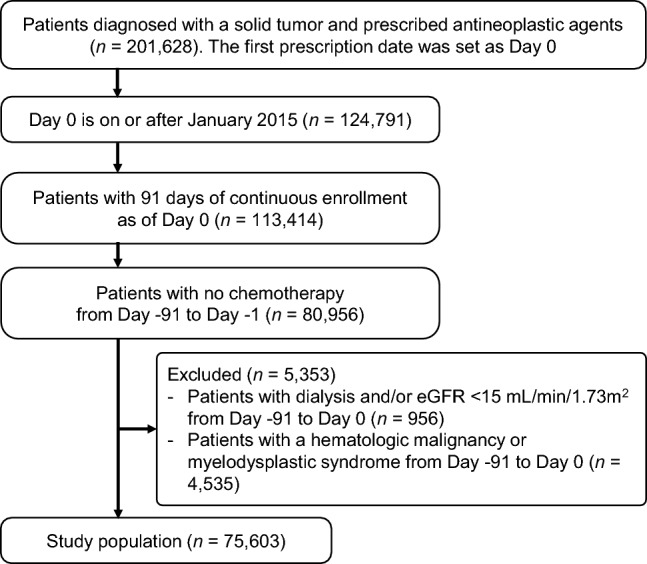
Flowchart of the study population

Data available until March 2025 were used to identify the eligible patients. Regarding patient selection (Fig. 1), patients diagnosed with a solid tumor and prescribed antineoplastic agents were extracted (*n* = 201,628), and the first prescription date was set as Day 0. Inclusion criteria were patients who had (i) Day 0 on or after January 2015, (ii) continuous 91-day enrollment as of Day 0, and (iii) no chemotherapy administration from Day -91 to -1. Exclusion criteria were patients with a record of dialysis, estimated glomerular filtration rate (eGFR) < 15 mL/min/1.73 m^2^, or a diagnosis of hematologic malignancy or myelodysplastic syndrome from Day -91 to 0.

### Evaluation

Patient demographics were collected, including age, gender, hospitalization status, surgical history, and radiation therapy history. Diagnoses of solid tumors were classified according to ICD-10 codes and divided into seven subgroups (stomach, colorectum, pancreas, lung, breast, uterus, and ovary) and others (Supplementary Table S1). Patients with multiple tumors were separately counted for each tumor. Antineoplastic agents were classified according to the WHO ATC classification (Supplementary Table S2). When multiple antineoplastic agents were administered, each class was counted separately.

**Fig. 2:**
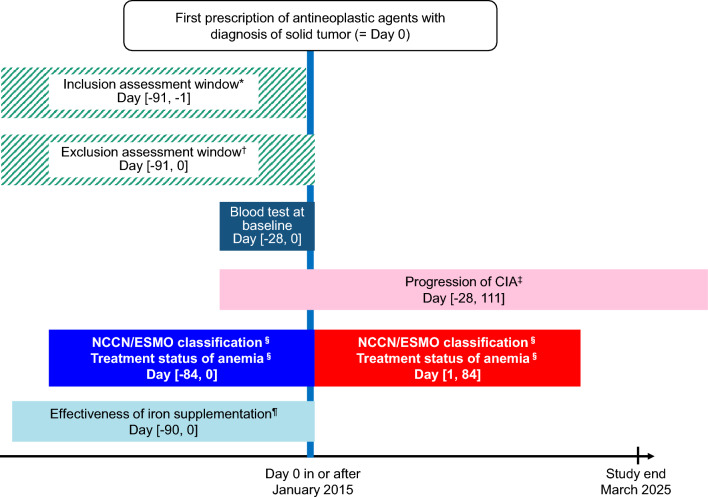
Study design. ^*^Continuous 91-day enrollment and no chemotherapy. ^†^Medical record of either dialysis, eGFR < 15 mL/min/1.73 m^2^, or diagnosis of hematologic malignancy or myelodysplastic syndrome. ^‡^Patients who did not receive treatment for anemia (iron preparation, ESA, or RBC transfusion), radiotherapy, or surgery from Day -91 to 0 were included. Data collected after death, the end of the study, or treatment during the follow-up period were excluded. ^§^Patients who had observational periods (Day -84 to 0 or Day 1 to 84) were included (i.e., excluding those who had the events of death, or study end during the observational period). Patients who did not receive treatment for anemia (iron preparation, ESA, or RBC transfusion), radiotherapy, or surgery from 30 days before starting iron supplementation to Day 0 were included. Data after death, study end, or treatment (excluding iron preparation) during the evaluation period were excluded

The study timeline is shown in Fig. 2. Blood test results were extracted for individual patients alongside the date, with the most recent result from Day -28 to 0 regarded as the baseline value (Day 0). Hb, TSAT, and ferritin levels were continuously monitored at three time points (i.e., 1 week [1W], 1 month [1 M], and 3 months [3 M]) after chemotherapy initiation). Representative days (and allowable ranges) for the three time points were defined as follows: 1W as Day 7 (Day 1 to 13), 1 M as Day 28 (Day 14 to 55), and 3 M as Day 84 (Day 56 to 111). If multiple values were present within a certain range, the value nearest to the representative day was used.

Anemia was defined and graded based on Hb values. Additionally, FID was defined as TSAT < 50% and ferritin ≥ 30 and ≤ 500 ng/mL according to NCCN guidelines, and as Hb ≥ 8 and < 10 with TSAT < 20% and ferritin ≥ 100 ng/mL according to ESMO guidelines [14, 15]. Other criteria are detailed in Supplementary Table S3).

### Anemia treatment

Prescriptions for oral iron, IV iron, ESA, and RBC transfusions were recorded (Supplementary Table S4). In Japan, three types of IV iron preparations are available, each with different maximum daily doses (saccharated ferric oxide [SFO], 120 mg; ferric carboxymaltose [FCM], 500 mg; and ferric derisomaltose [FDI], 1000 mg). Therefore, each preparation was examined separately. The total cumulative iron dose (mg) was calculated for oral and IV iron. Since medical procedure codes alone cannot specifically identify RBC transfusions (i.e., they may also include other types of transfusions), RBC transfusion was defined as the presence of transfusion procedure codes along with a recorded Hb level of < 10 g/dL within the preceding 3 days.

### Statistical analysis

Categorical variables are presented as numbers and proportions, and continuous data are summarized as medians with interquartile ranges (IQR). Missing values were not imputed for any variable. All analyses were performed using Python 3.12 (Python Software Foundation, Wilmington, DE, USA) and R version 4.3 (R Foundation for Statistical Computing, Vienna, Austria).

## Results

### Anemia management prior to chemotherapy initiation in Japan

Baseline demographics for the extracted 75,603 patients (median age: 71 years; females: 43.4%) are shown in Table 1. Over half of the patients were hospitalized on Day 0. The solid tumor types were colorectal (18.4%), lung (17.3%), breast (13.1%), stomach (10.5%), pancreas (7.1%), uterus (3.5%), ovary (3.0%), and others (Supplementary Table S1). Pyrimidine analogs (48.9%) and platinum-based agents (37.9%) were the most common antineoplastic agents prescribed (Supplementary Table S2). Prior surgery and radiation therapy between Day -91 and 0 were observed in 33.3% and 6.6% of patients, respectively.

**Table 1:** Baseline patient demographics

	Assessment period	*n* (%) <share %>	Median [IQR]
Study population	Day 0	75,603	
Age (year)	Day 0	75,603 (100.0)	71 [64–77]
Female gender	Day 0	32,782 (43.4)	
Hospitalization	Day 0	43,012 (56.9)	
Solid tumor type	See footnote^*^		
Stomach		7,936 (10.5)	
Colorectum		13,886 (18.4)	
Pancreas		5,386 (7.1)	
Lung		13,114 (17.3)	
Breast		9,890 (13.1)	
Uterus		2,674 (3.5)	
Ovary		2,305 (3.0)	
Antineoplastic agent^**^	Day 0–27		
Platinum-based		28,641 (37.9)	
Anthracycline		8,374 (11.1)	
Pyrimidine analog		36,955 (48.9)	
Taxane		13,628 (18.0)	
Immune checkpoint inhibitor		4,138 (5.5)	
Other		11,657 (15.4)	
History of surgery	Day -91–0	25,149 (33.3)	
History of radiation therapy	Day -91–0	4,961 (6.6)	
Hb (g/dL)	Day -28–0	72,085 (95.3)	12.2 [11.0–13.4]
<8		893 <1.2>	
≥8 and <10		7,099 <9.8>	
≥10 and <LLN		31,752 <44.0>	
≥LLN		32,341 <44.9>	
MCV (fL)	Day -28–0	72,048 (95.3)	91.8 [88.1–95.4]
<80		2,778 <3.9>	
≥80 and <100		63,566 <88.2>	
≥100		5,704 <7.9>	
Serum iron (μg/dL)	Day -28–0	6,639 (8.8)	55.0 [32.0–84.0]
TIBC (μg/dL)	Day -28–0	2,494 (3.3)	282.0 [233.0–332.0]
UIBC (μg/dL)	Day -28–0	3,237 (4.3)	219.0 [175.0–275.0]
TSAT^***^ (%)	Day -28–0	3,013 (4.0)	17.7 [10.0–28.1]
<20		1,715 <56.9>	
≥20 and <50		1,161 <38.5>	
≥50		137 <4.5>	
Serum ferritin (ng/mL)	Day -28–0	3,816 (5.0)	104.0 [32.0–271.8]
<30		899 <23.6>	
≥30 and <100		980 <25.7>	
≥100 and ≤500		1,510 <39.6>	
>500 and ≤800		206 <5.4>	
>800		221 <5.8>	
Serum vitamin B_12_ (pg/mL)	Day -28–0	583 (0.8)	527.0 [327.0–987.0]
≥200		542 <93.0>	
Serum folate (ng/mL)	Day -28–0	479 (0.6)	7.2 [4.9–10.9]
≥3		438 <91.4>	
CRP (mg/dL)	Day -28–0	65,030 (86.0)	0.2 [0.1–1.1]
≤0.5		42,368 <65.2>	
>0.5 and ≤1.0		6,147 <9.5>	
>1.0		16,515 <25.4>	

IQR, interquartile range; Hb, hemoglobin; MCV, mean corpuscular volume; TIBC, total iron-binding capacity, UIBC, unsaturated iron-binding capacity, TSAT, transferrin saturation, CRP, C-reactive protein

^*^The numbers of patients with solid tumors were counted during the month including Day 0 and the previous two months. Patients with multiple tumors were separately counted for each tumor type

^**^Patients with multiple antineoplastic agents were separately counted for each agent class

^***^TSAT was calculated using the data from the same day of either ‘serum iron and TIBC’ or ‘serum iron and UIBC’

**Fig. 3:**
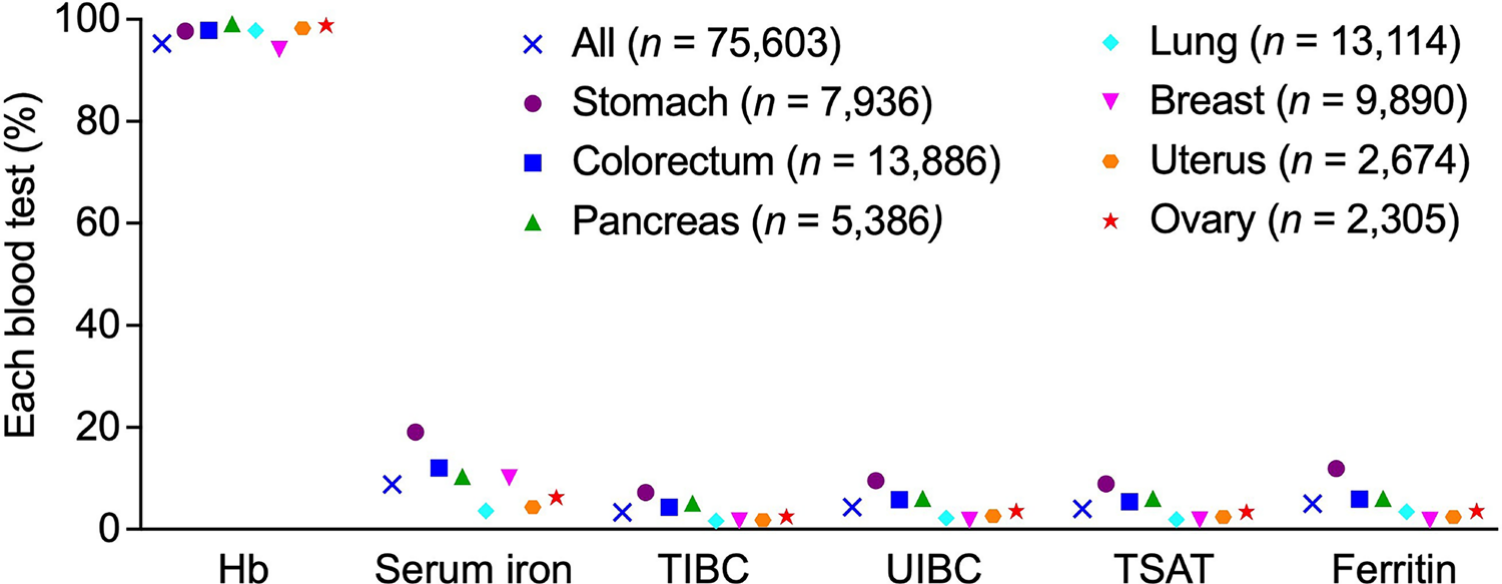
Blood test proportion at baseline. Values are proportions of patients in each tumor type. TSAT was calculated using data from the same day of either ‘serum iron and TIBC’ or ‘serum iron and UIBC’

Baseline Hb values were below the lower limit of normal (LLN) level in 55.1% of patients (males: < 13 g/dL; females: < 12 g/dL), while the proportion of patients with Hb < 8 g/dL and 8– < 10 g/dL were 1.2% and 9.8%, respectively. Serum iron, TIBC, UIBC, TSAT, and ferritin levels were measured in < 10% of patients. The proportions of detailed blood tests did not markedly differ across cancer types (Fig. 3; Supplementary Table S5). Low TSAT levels (< 20%) were observed in over half of all cancer types. Median ferritin levels were low in gastric cancer and colorectal cancer, at 48.9 ng/mL and 63.0 ng/mL, respectively (Supplementary Table S6).

### CIA progression

**Fig. 4:**
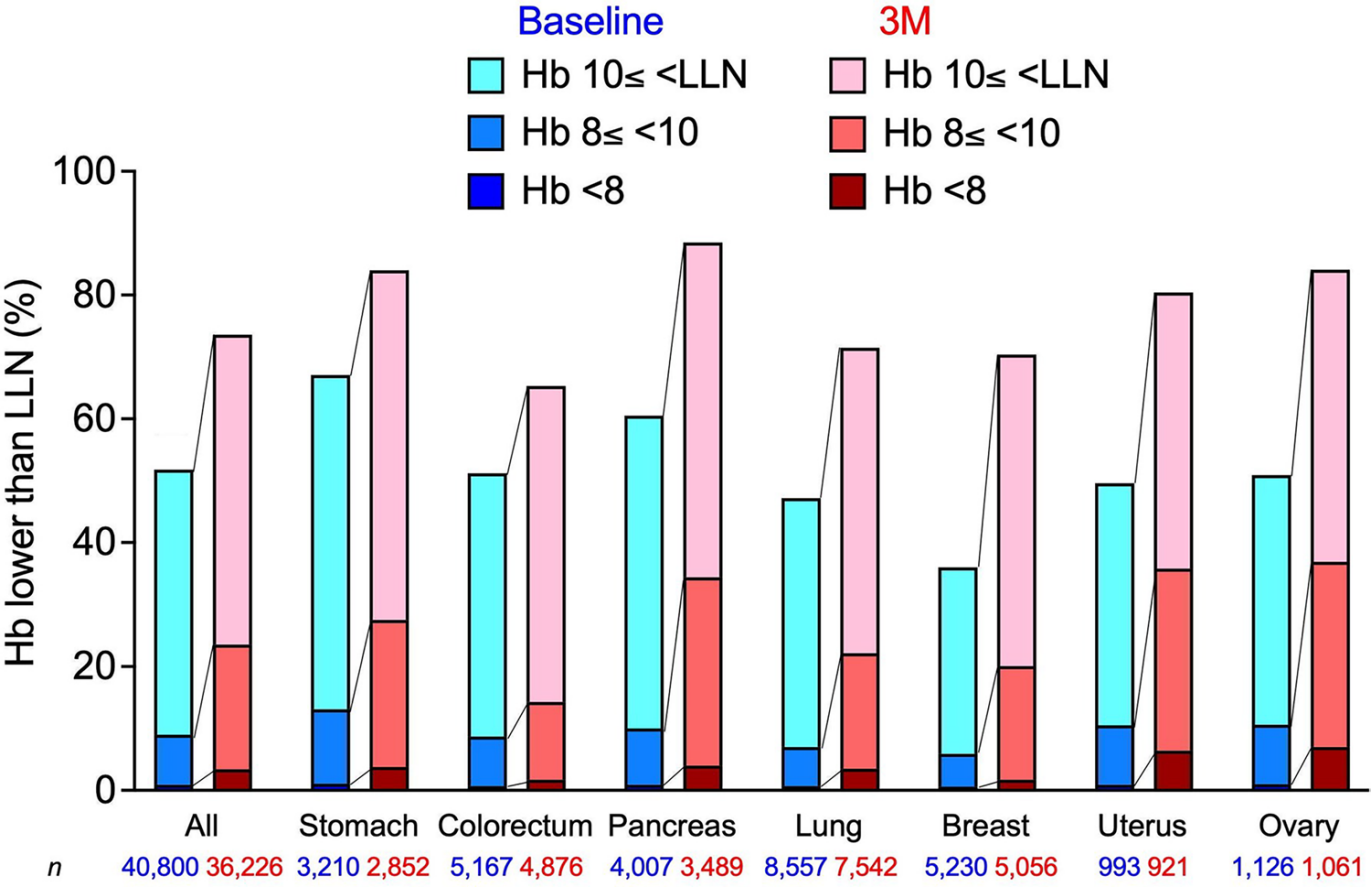
Anemic state based on Hb value classification before and after chemotherapy. Hb values are classified into 3 classes (10– < LLN, 8– < 10, and < 8 g/dL) and represented by stacked bars

To determine the effects of chemotherapy on anemia progression, we analyzed blood test results at baseline and 3 M (based on the determination that the CIA occurs frequently after starting chemotherapy three months later) in patients who had not received any anemia treatment (iron prescription, ESA, or RBC transfusion), radiation therapy, or surgical operation between Day -91 and 0. Across all tumor subgroups, the number of patients with Hb < LLN at 3 M increased markedly (Fig. 4). Hb levels gradually decreased from 1W, reaching their lowest value at 3 M (Supplementary Table S7). Median TSAT and ferritin levels were lower at baseline than at 3 M in most cancer types (Supplementary Tables S8– S9).

### NCCN/ESMO classification

**Fig. 5:**
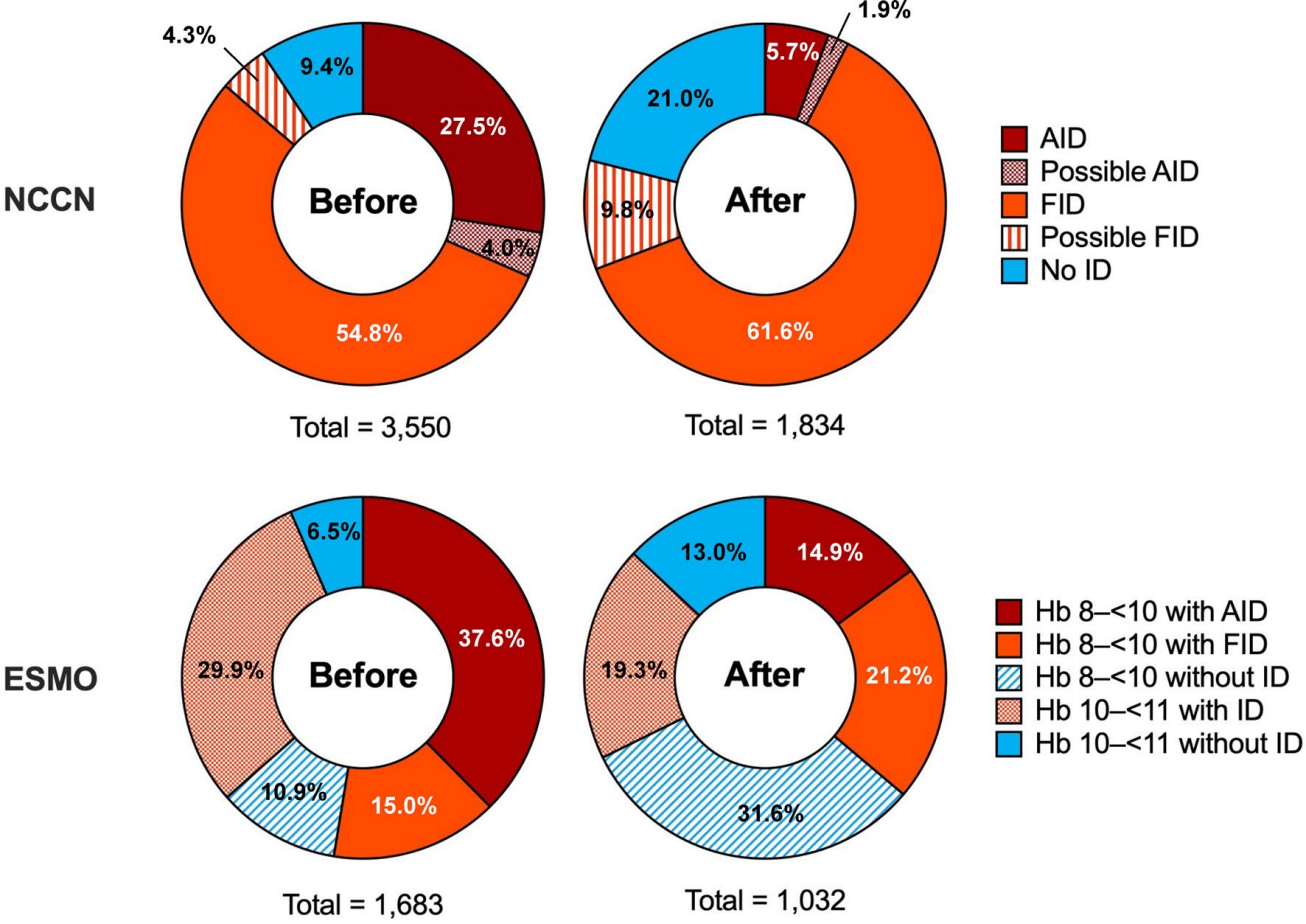
Proportion of anemia types according to the NCCN and ESMO guidelines before and after chemotherapy. These classifications were based on the TSAT, ferritin and Hb values (see Supplementary Table S3) obtained before and after chemotherapy initiation, from Day -84 to 0 (indicated as Before) and Day 1 to 84 (After), respectively. ‘Total’ shows patients who had all three laboratory values, determined by Hb and ferritin measured within ± 3 days of TSAT, in each period. ID, iron deficiency

Focusing on the periods before chemotherapy initiation (Day -84 to 0) and after chemotherapy initiation (Day 1 to 84), we classified patients who had three types of laboratory values (Hb, TSAT, and ferritin) into five categories based on the NCCN guidelines and five categories based on the ESMO guidelines (Fig. 5). According to the NCCN criteria, the proportions of patients with AID and FID before chemotherapy were 27.5% and 54.8%, respectively. In contrast, after chemotherapy initiation, the proportions were 5.7% and 61.6%, respectively. The eligible patients in this analysis had a higher prevalence of gastrointestinal malignancies, lower Hb levels (< 10 g/dL), lower mean corpuscular volume level (MCV; < 80 fL), and a history of surgery before chemotherapy than the whole population (Supplementary Table S11).

### Anemia treatment before and after chemotherapy

The proportion of patients prescribed oral or IV iron was approximately 8% and 3%, respectively, before chemotherapy (Day -84 to 0), and approximately 8% and 1%, respectively, after chemotherapy initiation (Day 1 to 84) (Table 2). The proportions of ESA and RBC transfusions showed no marked differences before and after chemotherapy initiation (approximately 0.3% and 4%, respectively). All types of IV iron were prescribed less frequently than oral iron, which was consistent across tumor subgroups (Supplementary Table S11).

**Table 2:** Anemia treatment before or after chemotherapy initiation

Group	Before				After			
	*n*	%	Iron dose (mg)		*n*	%	Iron dose (mg)	
			Median	[IQR]			Median	[IQR]
Total	67,332	100.0			62,045	100.0		
Oral iron	5,044	7.5	2,700	[1,100–5,200]	4,828	7.8	4,300	[1,890–8,400]
SFO	1,929	2.9	360	[200–600]	559	0.9	320	[160–640]
FCM	114	0.2	500	[500–1,000]	52	0.1	500	[500–1,000]
FDI	4	< 0.1	1,000	[750–1,250]	5	< 0.1	1,500	[1,000–1,500]
ESA	136	0.2			174	0.3		
RBC transfusion (Hb < 10)	2,982	4.4			2,302	3.7		

‘Total’ shows all patients during the observational period before starting chemotherapy (Day -84 to 0, shown as ‘Before’) or after starting chemotherapy (Day 1 to 84, shown as ‘After’), respectively. The iron dose represents the total cumulative iron dose during this period. Detailed data grouped by tumor type are presented in Supplementary Table S10

IQR, interquartile range; SFO, saccharated ferric oxide; FCM, ferric carboxymaltose; FDI, ferric derisomaltose; ESA, erythropoiesis-stimulating agent; RBC, red blood cell; Hb, hemoglobin

### Anemia parameter profile following iron supplementation before chemotherapy

**Fig. 6:**
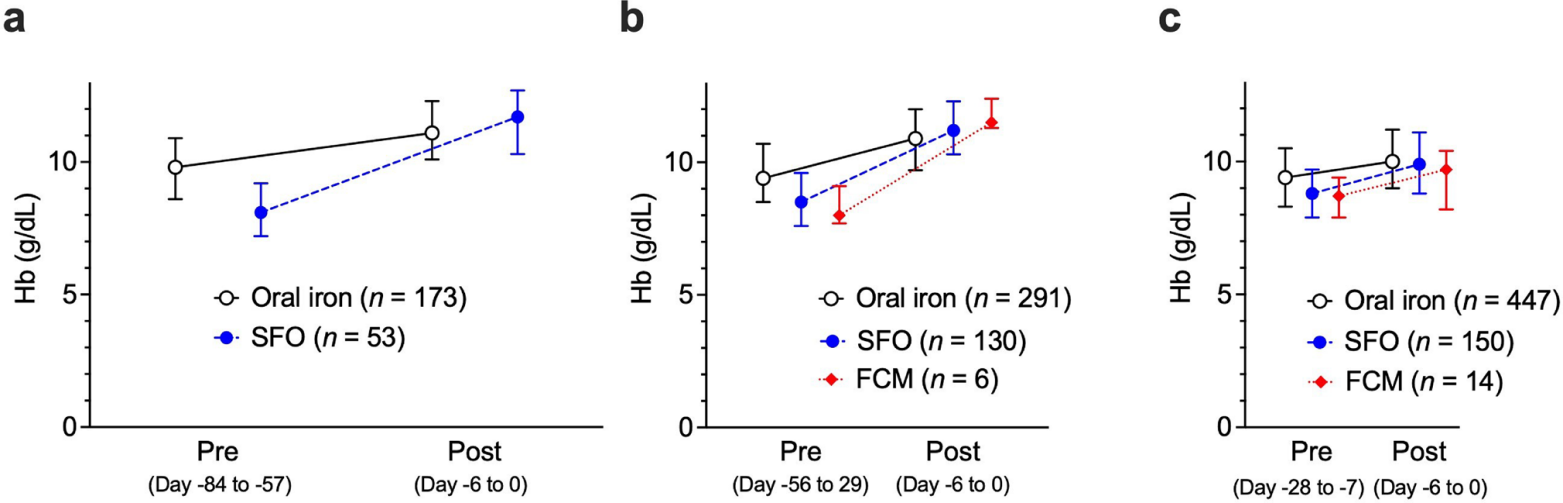
Changes in Hb values following iron supplementation before chemotherapy. Eligible patients in this analysis had both ‘Pre’ and ‘Post’ Hb values. Changes in Hb values are shown in median ± IQR (see Supplementary Table S12). Since panels **a**–**c** contain groups with small sample sizes, these results should be interpreted with caution. In particular, data for FCM group in panel **a** were omitted due to the limited sample size (*n* = 2)

To investigate the change in anemia parameters after iron supplementation in patients, we evaluated the changes in Hb levels after iron prescription (oral iron, SFO, or FCM) prior to chemotherapy initiation as a descriptive analysis, in which patients who received blood transfusions, ESA prescriptions, surgeries, or radiation therapy between 30 days before starting iron supplementation and Day 0 were excluded. The periods of iron treatment initiation were classified as Day -84 to -57 (8–12 weeks before), Day -56 to -29 (4–8 weeks before), and Day -28 to -7 (1–4 weeks before). Hb levels were evaluated twice, labelled as ‘Pre’ (6–0 days before iron supplementation) and ‘Post’ (immediately before chemotherapy initiation; Day -6 to 0) (Fig. 6). Hb levels increased across all groups following iron supplementation. Hb levels before iron therapy were lower in patients administered IV iron than in those administered oral iron. Patients in any iron groups started iron therapy just before chemotherapy initiation. Detailed data and dosing information are presented in Supplementary Table S12. Among patients who received iron therapy between Day -56 and -29, the pre- and post-therapy Hb levels were 8.0 g/dL and 11.5 g/dL in the FCM group, 8.5 g/dL and 11.2 g/dL in the SFO group, and 9.4 g/dL and 10.9 g/dL in the oral iron group. In patients who began iron therapy between Day -28 and Day -7, the median start date was Day -13.5 in the FCM group and Day -17.5 in the SFO group. Regardless of the timing, the SFO group had a higher proportion of patients receiving concomitant oral iron therapy than the FCM group. Notably, no patients in this study were prescribed FDI.

## Discussion

This study utilized Japanese RWD to examine the current state of anemia management, focusing on iron-deficiency anemia in patients with solid tumors before and after starting chemotherapy. We demonstrated that over half of the patients had anemia characterized by low Hb levels before and after chemotherapy, with its prevalence increasing after chemotherapy initiation, regardless of cancer type. In this study, the number of patients gradually decreased because data after events such as death during follow-up were excluded; therefore, there may potentially be more CIA patients in reality. On the other hand, iron evaluation was not widely performed in these patients during this period, as evidenced by the low proportion (< 10%) of patients tested for iron-related markers. Previous reports have shown that TSAT and ferritin levels are not widely measured in European countries [24, 25]. Our findings revealed the frequency of iron assessment in Japan is even lower compared to Europe, potentially due to the absence of clinical practice guidelines for CIA in Japan, resulting in insufficient awareness of the need for blood testing before and after chemotherapy initiation. CIA is known to affect patient quality of life (QOL), continuation of chemotherapy, and prognosis [26–28]. Although anemia that occurs during chemotherapy is often thought to be caused by bone marrow suppression, further blood examinations, including iron-related markers, should be performed for the appropriate diagnosis and treatment of CIA.

Generally, patients undergoing iron-related marker testing are presumed to have suspected iron deficiency anemia at the time of measurement. In this study, among patients who had Hb, TSAT, and ferritin results (< 10% of the total population), a slightly higher proportion of patients with moderate to severe anemia (Hb < 10 g/dL), microcytic anemia (MCV < 80 fL), a history of surgery prior to chemotherapy, or gastrointestinal malignancies was observed, compared to the overall population. To capture these patient characteristics in more detail, prospective studies are warranted that consider various factors influencing hematopoietic efficiency, such as difference of age distribution across tumor types, comprehensive nutritional status, and surgical extent of resection, which was not fully assessed in this study. Among patients with available iron-status data in our study, over half met the NCCN FID criteria (TSAT < 50% with ferritin ≥ 30 and ≤ 500 ng/mL) both before and after chemotherapy initiation, with the proportion increasing after initiation. While NCCN and ESMO guidelines [14, 15] states ESAs, RBC transfusions and iron therapy as treatment options for CIA with iron deficiency, ESAs are not approved in Japan because of concerns about risks such as worsening prognosis and promoting tumor proliferation [29]. Furthermore, blood product use is increasingly discouraged given the supply–demand balance is at risk of disruption due to the declining birthrate and aging population [30, 31]. In this clinical context, IV iron administration is recommended for patients with iron deficiency, particularly those with FID, taking into account that oral iron is less effective than IV iron. However, similar to previous international research [32], we found a tendency toward lower proportions of IV iron prescriptions. Our findings increase clinical awareness of anemia, including FID, and reaffirm the critical need for proper diagnosis and therapeutic intervention before and after chemotherapy.

Among patients who received iron therapy before chemotherapy initiation, those who received IV iron 4–12 weeks before initiation (Fig. 6a and b) had lower Hb levels than those who received oral iron and greater Hb level improvement. Although the number of patients receiving iron therapy increased closer to chemotherapy initiation, data from the 4–8 weeks pretreatment period (Fig. 6b) suggested that FCM, a high-dose IV iron therapy, tended to elevate Hb levels, even in patients with low Hb levels. However, the results may be confounded by the severity of anemia and other clinical factors. In addition, this study could not evaluate clinically relevant outcomes in patients receiving iron therapy, such as the onset of anemia after chemotherapy, the requirement for transfusion, chemotherapy continuation and survival rates. Therefore, further studies incorporating prospective designs or comparative analysis are necessary to confirm the efficacy of high-dose IV therapy for CIA with various perspectives. In general, high-dose IV iron facilitates the administration of an optimal dose with fewer outpatient visits and vascular punctures than low-dose IV iron, making it suitable for severe anemia and rapid Hb improvement [33]. Another RWD study of patients prescribed iron preparations in Japan also showed that FCM, rather than SFO, was more likely to be administered to patients with lower Hb levels [34]. In addition, this previous study demonstrated that patients receiving high-dose IV iron at an appropriate dose achieved sufficient Hb improvement. However, as previously reported [35, 36], even with IV iron therapy, Hb levels may take up to a month or longer to sufficiently increase, depending on the patient’s condition and dose. Therefore, if iron supplementation is initiated immediately before chemotherapy, patients may begin treatment while Hb levels remain low, limiting the efficacy of the iron preparations. To address this, laboratory values should be continuously monitored after chemotherapy and the need for iron supplementation should be reassessed.

This study had some limitations. First, this retrospective study utilized RWD collected from contracted medical institutions, which may not fully represent clinical practices across Japan. Second, patients could not be followed up after transfer to other healthcare facilities, which may have led to an underestimation of diagnosis, medications, or medical procedures, including laboratory testing.

In conclusion, the prevalence of anemia increased following the initiation of treatment in patients with solid tumors undergoing chemotherapy in Japan, regardless of cancer type. Despite a tendency toward infrequent measurement of iron parameters, FID was frequently observed among patients for whom these data were available. Our findings suggest the importance of continuous monitoring iron parameters and appropriate therapeutic intervention for anemia before and after initiating chemotherapy.

## Data availability statement

The data used in this study are not publicly available because they were obtained from HCEI and JMDC Inc., but are available from the corresponding author with the permission of HCEI and JMDC Inc. upon reasonable request.

## Electronic Supplementary Material

Below is the link to the electronic supplementary material.

Supplementary Material 1
