# Supplementary material for: Management of iron deficiency anemia and the role of intravenous iron supplementation in patients undergoing cancer chemotherapy: a real-world retrospective study in Japan
Source: Int J Clin Oncol. 2026 Jun 2;31(7):1408–17. doi: 10.1007/s10147-026-03057-4 (PMC13303690; doi:10.1007/s10147-026-03057-4)
Supplement: Supplementary file 1 — Supplementary Material 1 [file 10147_2026_3057_MOESM1_ESM.pdf]

Supplementary information

Management of iron deficiency anemia and the role of intravenous iron supplementation in patients undergoing cancer chemotherapy: A real-world retrospective study in Japan

Supplementary Table S1 Definition of solid tumor and the number of patients
Supplementary Table S2 Definition of antineoplastic agents and the number of patients
Supplementary Table S3 Definition of anemia types by NCCN and ESMO guidelines
Supplementary Table S4 Definition of antianemic treatments
Supplementary Table S5 Proportion of blood test at baseline (grouped by tumor type)
Supplementary Table S6 Baseline demographics (grouped by tumor type)
Supplementary Table S7 Hb values after starting chemotherapy (grouped by tumor type)
Supplementary Table S8 TSAT values after starting chemotherapy (grouped by tumor type)
Supplementary Table S9 Ferritin values after starting chemotherapy (grouped by tumor type)
Supplementary Table S10 Baseline demographics (eligible population of NCCN/ESMO classification)
Supplementary Table S11 Treatment of anemia before or after starting chemotherapy (grouped by tumor type)
Supplementary Table S12 Effects of pretreatment with iron prior to chemotherapy on Hb values

Supplementary Table S1 Definition of solid tumor and the number of patients

Solid tumor	ICD-10 code	*n*	%
Lip, oral cavity, and pharynx	C00-C14	1,762	2.3%
Esophagus	C15	2,920	3.9%
Stomach	C16	7,936	10.5%
Colorectum	C18–C20	13,886	18.4%
Colon	C18	9,608	12.7%
Rectum (including rectosigmoidal junction)	C19–C20	5,667	7.5%
Liver and intrahepatic bile duct	C22	3,856	5.1%
Gallbladder and bile duct	C23–C24	2,101	2.8%
Pancreas	C25	5,386	7.1%
Larynx	C32	577	0.8%
Lung (including trachea and bronchi)	C33–C34	13,114	17.3%
Skin	C43–C44	432	0.6%
Breast	C50	9,890	13.1%
Uterus	C53–C55	2,674	3.5%
Cervix uteri	C53	1,055	1.4%
Corpus uteri	C54	1,667	2.2%
Ovary (including fallopian tube and peritoneum)	C48.2, C56, C57.0	2,305	3.0%
Prostate	C61	3,837	5.1%
Kidney and other urinary organs	C64–C66, C68	3,013	4.0%
Bladder	C67	6,142	8.1%
Thyroid gland	C73	526	0.7%
Brain and central nervous system	C70–C72, C75.1–C75.3	567	0.7%
Others	C17, C21, C26, C30, C31, C37–C41, C45–C47, C48.0–C48.1, C49, C51, C52, C55, C57.9, C58, C60, C62, C63, C69, C74, C75.0, C75.4–C75.9, C76	11,657	15.4%

Patients with multiple tumors were separately counted for each tumor type. Rows with red shadow indicate solid tumors highlighted in subgroup analysis. In the exclusion criteria, hematologic malignancy and myelodysplastic syndrome were defined by ICD-10 code C81–C96 and D46, respectively.

Supplementary Table S2 Definition of antineoplastic agents and the number of patients

Antineoplastic agent	WHO ATC code	*n*	%
Nitrogen mustard analogues	L01AA	4,730	6.3%
Alkyl sulfonates	L01AB	1	<0.1%
Ethylene imines	L01AC	1	<0.1%
Nitrosoureas	L01AD	47	0.1%
Other alkylating agents	L01AX	475	0.6%
Folic acid analogues	L01BA	1,849	2.4%
Pyrimidine analogues	L01BC	36,955	48.9%
Vinca alkaloids and analogues	L01CA	1,083	1.4%
Podophyllotoxin derivatives	L01CB	1,902	2.5%
Taxanes	L01CD	13,628	18.0%
Topoisomerase 1 (TOP1) inhibitors	L01CE	1,997	2.6%
Other plant alkaloids and natural products	L01CX	1,115	1.5%
Actinomycines	L01DA	13	<0.1%
Anthracyclines and related substances	L01DB	8,374	11.1%
Other cytotoxic antibiotics	L01DC	876	1.2%
BCR-ABL tyrosine kinase inhibitors	L01EA	414	0.5%
Epidermal growth factor receptor (EGFR) tyrosine kinase inhibitors	L01EB	1,899	2.5%
B-Raf serine-threonine kinase (BRAF) inhibitors	L01EC	42	0.1%
Anaplastic lymphoma kinase (ALK) inhibitors	L01ED	180	0.2%
Mitogen-activated protein kinase (MEK) inhibitors	L01EE	40	0.1%
Cyclin-dependent kinase (CDK) inhibitors	L01EF	1,089	1.4%
Mammalian target of rapamycin (mTOR) kinase inhibitors	L01EG	232	0.3%
Human epidermal growth factor receptor 2 (HER2) tyrosine kinase inhibitors	L01EH	19	<0.1%
Vascular endothelial growth factor receptor (VEGFR) tyrosine kinase inhibitors	L01EK	182	0.2%
Fibroblast growth factor receptor (FGFR) tyrosine kinase inhibitors	L01EN	0	0.0%
Other protein kinase inhibitors	L01EX	2,345	3.1%
HER2 (Human Epidermal Growth Factor Receptor 2) inhibitors	L01FD	2,238	3.0%
EGFR (Epidermal Growth Factor Receptor) inhibitors	L01FE	1,332	1.8%
PD-1/PD-L1 (Programmed cell death protein 1/death ligand 1) inhibitors	L01FF	4,138	5.5%
VEGF/VEGFR (Vascular Endothelial Growth Factor) inhibitors	L01FG	4,887	6.5%
Other monoclonal antibodies and antibody drug conjugates	L01FX	348	0.5%
Combinations of monoclonal antibodies and antibody drug conjugates	L01FY	33	<0.1%
Platinum compounds	L01XA	28,641	37.9%
Methylhydrazines	L01XB	9	<0.1%
Sensitizers used in photodynamic/radiation therapy	L01XD	254	0.3%
Poly (ADP-ribose) polymerase (PARP) inhibitors	L01XK	49	0.1%
Other antineoplastic agents	L01XX	1,827	2.4%

Patients with multiple antineoplastic agents were separately counted for each agent class.

Supplementary Table S3 Definition of anemia types by NCCN and ESMO guidelines

Guideline	Classification	Definition
NCCN	AID	TSAT <20% with ferritin <30 ng/mL
	Possible AID	TSAT ≥20% and <50% with ferritin <30 ng/mL
	FID	TSAT <50% with ferritin ≥30 and ≤500 ng/mL
	Possible FID	TSAT <50% with ferritin >500 and ≤800 ng/mL
	No ID	TSAT ≥50% or ferritin >800 ng/mL
ESMO	Hb 8–<10 with AID	Hb ≥8 and <10 with ferritin <100 ng/mL
	Hb 8–<10 with FID	Hb ≥8 and <10 with TSAT <20% and ferritin ≥100 ng/mL
	Hb 8–<10 without ID	Hb ≥8 and <10 with TSAT ≥20% and ferritin ≥100 ng/mL
	Hb 10–<11 with ID	Hb ≥10 and <11 with TSAT <20% or ferritin <100 ng/mL
	Hb 10–<11 without ID	Hb ≥10 and <11 with TSAT ≥20% or ferritin ≥100 ng/mL

ID, iron deficiency. TSAT was calculated using the data from the same day of either 'serum iron and TIBC' or 'serum iron and UIBC'.

Supplementary Table S4 Definition of antianemic treatments

Category	Agent	WHO ATC code (Japanese-specific code)
Oral iron	Ferrotrenine	B03AA (613220025)
	Ferrous fumarate	B03AA02
	Ferrous sulfate	B03AA07
	Sodium ferrous citrate	B03AA12
	Ferric pyrophosphate, soluble	B03AB (613220036, 620005924, 1000001252)
	Ferric citrate hydrate	V03AE08
SFO	Saccharated ferric oxide	B03AC (620000257, 620005208, 620006266, 643220003, 643220006)
	Cideferron	B03AC (620004747, 643220004)
	Sodium chondroitin sulfate ester and iron colloid	B03AC (640406008, 643220005)
	Ferric gluconate	B03AC (643220002)
FCM	Ferric carboxymaltose	B03AC (622676601)
FDI	Ferric derisomaltose	B03AC (622926501, 622926601)
ESA	Erythropoietin	B03XA01
	Darbepoetin alfa	B03XA02
	Epoetin beta pegol	B03XA03

RBC transfusion was defined as the presence of transfusion procedure codes in K920 along with a recorded Hb level of <10 g/dL within the preceding 3 days.

Supplementary Table S5 Proportion of blood test at baseline (grouped by tumor type)

Blood test<br>*n* (%)	Hb	Serum iron	TIBC	UIBC	TSAT	Ferritin
All	72,085 (95.3)	6,639 (8.8)	2,494 (3.3)	3,237 (4.3)	3,013 (4.0)	3,816 (5.0)
Stomach	7,751 (97.7)	1,518 (19.1)	568 (7.2)	754 (9.5)	707 (8.9)	941 (11.9)
Colorectum	13,600 (97.9)	1,666 (12.0)	593 (4.3)	799 (5.8)	756 (5.4)	825 (5.9)
Pancreas	5,341 (99.2)	562 (10.4)	275 (5.1)	329 (6.1)	328 (6.1)	329 (6.1)
Lung	12,825 (97.8)	466 (3.6)	210 (1.6)	285 (2.2)	255 (1.9)	451 (3.4)
Breast	9,304 (94.1)	998 (10.1)	155 (1.6)	170 (1.7)	176 (1.8)	171 (1.7)
Uterus	2,628 (98.3)	114 (4.3)	47 (1.8)	70 (2.6)	63 (2.4)	63 (2.4)
Ovary	2,279 (98.9)	146 (6.3)	57 (2.5)	84 (3.6)	78 (3.4)	84 (3.6)

TSAT was calculated using the data from the same day of either 'serum iron and TIBC' or 'serum iron and UIBC'.

Supplementary Table S6 Baseline demographics (grouped by tumor type)

	Stomach (*N* = 7,936)*				Colorectum (*N* = 13,886)*				Pancreas (*N* = 5,386)*				Lung (*N* = 13,114)*				Breast (*N* = 9,890)*				Uterus (*N* = 2,674)*				Ovary (*N* = 2,305)*			
	n	%	Median	IQR	*n*	%	Median	IQR	*n*	%	Median	IQR	*n*	%	Median	IQR	*n*	%	Median	IQR	*n*	%	Median	IQR	*n*	%	Median	IQR
Age (year)			73	67–78			71	64–77			73	67–78			73	67–78			63	52–71			62	52–71			63	53–72
Female gender	2,004	25.3			5,424	39.1			2,288	42.5			3,972	30.3			9,823	99.3			2,670	99.9			2,297	99.7		
Hospitalization	3,446	43.4			5,221	37.6			3,118	57.9			10,226	78.0			3,139	31.7			1,937	72.4			1,611	69.9		
Antineoplastic agent**																												
Platinum-based	3,250	40.9			6,666	48.0			618	11.5			6,498	49.5			461	4.7			2,295	85.8			2,003	86.9		
Anthracycline	152	1.9			207	1.5			40	0.7			243	1.8			3,149	31.8			237	8.9			111	4.8		
Pyrimidine analog	6,509	82.0			12,511	90.1			5,162	95.8			2,321	17.7			2,625	26.5			196	7.3			152	6.6		
Taxane	680	8.6			337	2.4			1,902	35.3			2,604	19.9			2,683	27.1			1,660	62.1			1,845	80.0		
Immune checkpoint inhibitor	375	4.7			142	1.0			16	0.3			2,151	16.4			93	0.9			36	1.3			6	0.3		
Others	566	7.1			610	4.4			112	2.1			3,190	24.3			2,394	24.2			127	4.8			99	4.3		
History of radiation therapy	233	2.9			350	2.5			141	2.6			1,325	10.1			880	8.9			336	12.6			28	1.2		
History of surgery	3,407	42.9			7,397	53.3			995	18.5			2,880	22.0			3,533	35.7			1,204	45.0			993	43.1		
Hb (g/dL)	7,751	97.7	11.6	10.3–12.8	13,600	97.9	12.1	10.9–13.2	5,341	99.2	11.9	10.8–13.0	12,825	97.8	12.7	11.5–13.8	9,304	94.1	12.4	11.5–13.2	2,628	98.3	11.9	10.8–12.8	2,279	98.9	11.8	10.7–12.7
<8	175	2.3			132	1.0			63	1.2			96	0.7			54	0.6			35	1.3			30	1.3		
≥8 and <10	1,253	16.2			1,539	11.3			593	11.1			850	6.6			529	5.7			293	11.1			272	11.9		
≥10 and <LLN	4,300	55.5			6,492	47.7			2,806	52.5			5,210	40.6			2,840	30.5			1,069	40.7			948	41.6		
≥LLN	2,023	26.1			5,437	40.0			1,879	35.2			6,669	52.0			5,881	63.2			1,231	46.8			1,029	45.2		
MCV (fL)	7,753	97.7	91.2	86.8–95.3	13,594	97.9	90.6	86.1–94.6	5,343	99.2	92.2	89.1–95.4	12,823	97.8	92.0	88.7–95.3	9,288	93.9	91.4	88.3–94.6	2,627	98.2	90.8	87.4–94.0	2,278	98.8	91.0	87.8–94.1
<80	512	6.6			1,050	7.7			81	1.5			251	2.0			279	3.0			109	4.1			68	3.0		
≥80 and <100	6,624	85.4			11,767	86.6			4,880	91.3			11,601	90.5			8,548	92.0			2,410	91.7			2,088	91.7		
≥100	617	8.0			777	5.7			382	7.1			971	7.6			461	5.0			108	4.1			122	5.4		
Serum iron (μg/dL)	1,518	19.1	47.0	26.0–78.0	1,666	12.0	52.0	31.0–82.0	562	10.4	54.3	34.0–77.0	466	3.6	47.0	26.0–76.0	998	10.1	70.0	49.0–95.0	114	4.3	46.0	25.0–65.0	146	6.3	33.5	21.0–58.0
TIBC (μg/dL)	568	7.2	290.5	249.0–342.0	593	4.3	306.0	254.0–351.0	275	5.1	264.0	230.0–309.0	210	1.6	238.5	200.0–293.0	155	1.6	315.0	263.0–365.0	47	1.8	273.0	213.0–344.0	57	2.5	240.0	200.0–272.0
UIBC (μg/dL)	754	9.5	238.0	185.0–294.0	799	5.8	237.0	191.0–296.0	329	6.1	203.0	166.0–246.0	285	2.2	202.0	159.0–248.0	170	1.7	236.5	190.0–307.0	70	2.6	222.5	178.0–289.0	84	3.6	194.0	157.0–234.5
TSAT (%)***	707	8.9	14.7	7.7–25.6	756	5.4	16.6	9.4–27.1	328	6.1	19.9	11.9–29.1	255	1.9	14.6	10.2–23.3	176	1.8	19.2	11.3–29.5	63	2.4	14.6	10.1–21.2	78	3.4	13.3	9.1–21.7
<20	454	64.2			452	59.8			166	50.6			172	67.5			92	52.3			43	68.3			54	69.2		
≥20 and <50	222	31.4			266	35.2			153	46.6			71	27.8			76	43.2			19	30.2			24	30.8		
≥50	31	4.4			38	5.0			9	2.7			12	4.7			8	4.5			1	1.6			0	0.0		
Serum ferritin (ng/mL)	941	11.9	48.9	17.9–154.8	825	5.9	63.0	24.6–179.8	329	6.1	180.7	70.4–396.2	451	3.4	191.8	70.3–395.4	171	1.7	77.7	31.2–190.0	63	2.4	106.9	45.0–290.0	84	3.6	193.5	79.9–400.5
<30	358	38.0			243	29.5			44	13.4			54	12.0			41	24.0			12	19.0			7	8.3		
≥30 and <100	263	27.9			267	32.4			62	18.8			89	19.7			61	35.7			19	30.2			18	21.4		
≥100 and ≤500	275	29.2			273	33.1			169	51.4			225	49.9			53	31.0			25	39.7			46	54.8		
>500 and ≤800	23	2.4			26	3.2			27	8.2			34	7.5			4	2.3			4	6.3			4	4.8		
>800	22	2.3			16	1.9			27	8.2			49	10.9			12	7.0			3	4.8			9	10.7		
Serum vitamin B_{12} (pg/mL)	219	2.8	482.0	286.0–905.0	69	0.5	447.0	342.0–987.0	40	0.7	897.0	517.5–1442.0	105	0.8	510.0	299.0–1025.0	24	0.2	548.0	327.0–796.0	11	0.4	422.0	356.0–793.0	15	0.7	485.0	434.0–796.0
≥200	201	91.8			66	95.7			39	97.5			93	88.6			24	100.0			11	100.0			15	100.0		
Serum folate (ng/mL)	155	2.0	7.4	5.5–11.1	55	0.4	6.9	4.5–12.4	36	0.7	7.8	5.4–10.3	87	0.7	7.7	4.6–10.7	19	0.2	7.2	4.6–13.7	10	0.4	6.9	4.1–14.4	15	0.7	6.1	5.4–11.6
≥3	143	92.3			49	89.1			35	97.2			76	87.4			17	89.5			9	90.0			15	100.0		
CRP (mg/dL)	7,271	91.6	0.2	0.1–0.9	12,684	91.3	0.2	0.1–0.6	5,226	97.0	0.3	0.1–1.3	12,489	95.2	0.4	0.1–2.0	6,324	63.9	0.1	0.1–0.4	2,282	85.3	0.2	0.1–0.8	1,983	86.0	0.3	0.1–1.5
≤0.5	4,826	66.4			9,235	72.8			3,227	61.7			6,804	54.5			5,116	80.9			1,582	69.3			1,219	61.5		
>0.5 and ≤1.0	713	9.8			1,040	8.2			554	10.6			1,319	10.6			439	6.9			208	9.1			172	8.7		
>1.0	1,732	23.8			2,409	19.0			1,445	27.7			4,366	35.0			769	12.2			492	21.6			592	29.9		

*The numbers of patients with solid tumors were counted during the month including Day 0 and the previous two months. Patients with multiple tumors were separately counted for each tumor type.

**Patients with multiple antineoplastic agents were separately counted for each agent class.

***TSAT was calculated using the data from the same day of either 'serum iron and TIBC' or 'serum iron and UIBC'.

Supplementary Table S7 Hb values after starting chemotherapy (grouped by tumor type)

		Time point	Baseline	1W	1M	3M
All		*n*	40,800	28,690	39,248	36,226
		Hb (g/dL)	12.4 [11.2-13.5]	12.0 [10.7-13.2]	11.7 [10.5-12.8]	***11.3*** [10.0-12.6]
	n (%)	<8	360 (0.9)	527 (1.8)	830 (2.1)	1,243 (3.4)
		8≤ <10	3,318 (8.1)	3,557 (12.4)	5,623 (14.3)	7,279 (20.1)
		10≤ <LLN	17,440 (42.8)	13,674 (47.7)	20,031 (51.0)	18,134 (50.1)
		LLN≤	19,682 (48.2)	10,932 (38.1)	12,764 (32.5)	9,570 (26.4)
Stomach		*n*	3,210	2,076	3,110	2,852
		Hb (g/dL)	11.9 [10.7–13.1]	11.6 [10.3–12.8]	11.3 [10.2–12.5]	***11.0*** [9.8–12.2]
	n (%)	<8	36 (1.1)	56 (2.7)	64 (2.1)	109 (3.8)
		8≤ <10	386 (12.0)	329 (15.8)	596 (19.2)	677 (23.7)
		10≤ <LLN	1,735 (54.0)	1,121 (54.0)	1,814 (58.3)	1,613 (56.6)
		LLN≤	1,053 (32.8)	570 (27.5)	636 (20.4)	453 (15.9)
Colorectum		*n*	5,167	2,548	5,071	4,876
		Hb (g/dL)	12.5 [11.3–13.7]	12.1 [10.9–13.3]	12.2 [10.9–13.3]	***11.9*** [10.7–13.0]
	n (%)	<8	34 (0.7)	44 (1.7)	62 (1.2)	81 (1.7)
		8≤ <10	413 (8.0)	264 (10.4)	527 (10.4)	613 (12.6)
		10≤ <LLN	2,194 (42.5)	1,199 (47.1)	2,399 (47.3)	2,486 (51.0)
		LLN≤	2,526 (48.9)	1,041 (40.9)	2,083 (41.1)	1,696 (34.8)
Pancreas		*n*	4,007	3,380	3,860	3,489
		Hb (g/dL)	12.1 [11.0–13.1]	11.5 [10.4–12.6]	11.1 [10.1–12.2]	***10.6*** [9.5–11.7]
	n (%)	<8	35 (0.9)	68 (2.0)	96 (2.5)	141 (4.0)
		8≤ <10	364 (9.1)	522 (15.4)	788 (20.4)	1,062 (30.4)
		10≤ <LLN	2,023 (50.5)	1,893 (56.0)	2,273 (58.9)	1,888 (54.1)
		LLN≤	1,585 (39.6)	897 (26.5)	703 (18.2)	398 (11.4)
Lung		*n*	8,557	7,370	8,240	7,542
		Hb (g/dL)	12.7 [11.5–13.8]	12.3 [11.0–13.4]	11.9 [10.7–13.1]	***11.5*** [10.1–12.8]
	n (%)	<8	62 (0.7)	107 (1.4)	163 (2.0)	267 (3.5)
		8≤ <10	536 (6.3)	732 (9.9)	1,061 (12.9)	1,401 (18.6)
		10≤ <LLN	3,442 (40.2)	3,436 (46.6)	4,213 (51.1)	3,727 (49.4)
		LLN≤	4,517 (52.8)	3,095 (42.0)	2,803 (34.0)	2,147 (28.5)
Breast		*n*	5,230	2,241	5,191	5,056
		Hb (g/dL)	12.4 [11.5–13.3]	12.0 [10.9–12.8]	11.7 [10.7–12.5]	***11.2*** [10.2–12.2]
	n (%)	<8	29 (0.6)	31 (1.4)	69 (1.3)	87 (1.7)
		8≤ <10	278 (5.3)	234 (10.4)	547 (10.5)	933 (18.4)
		10≤ <LLN	1,575 (30.1)	853 (38.1)	2,442 (47.0)	2,543 (50.3)
		LLN≤	3,348 (64.0)	1,123 (50.1)	2,133 (41.1)	1,493 (29.5)
Uterus		*n*	993	798	974	921
		Hb (g/dL)	12.0 [10.9–13.0]	11.7 [10.6–12.7]	11.2 [10.2–12.2]	***10.5*** [9.4–11.6]
	n (%)	<8	9 (0.9)	14 (1.8)	32 (3.3)	59 (6.4)
		8≤ <10	95 (9.6)	95 (11.9)	167 (17.1)	271 (29.4)
		10≤ <LLN	388 (39.1)	346 (43.4)	481 (49.4)	411 (44.6)
		LLN≤	501 (50.5)	343 (43.0)	294 (30.2)	180 (19.5)
Ovary		*n*	1,126	917	1,112	1,061
		Hb (g/dL)	11.9 [10.9–12.8]	11.5 [10.4–12.4]	11.1 [10.1–12.0]	***10.6*** [9.4–11.5]
	n (%)	<8	11 (1.0)	20 (2.2)	34 (3.1)	74 (7.0)
		8≤ <10	108 (9.6)	127 (13.8)	218 (19.6)	317 (29.9)
		10≤ <LLN	454 (40.3)	429 (46.8)	569 (51.2)	501 (47.2)
		LLN≤	553 (49.1)	341 (37.2)	291 (26.2)	169 (15.9)

Values are medians with IQRs, and the minimum values within each tumor type are indicated in boldface italics. LLN stands for the lower limit of normal (male: 13 g/dL, female: 12 g/dL). These data were taken from patients who did not receive treatment for anemia (iron preparation, ESA, or RBC transfusion), radiotherapy, or surgery from Day -91 to 0. Laboratory values were continuously monitored at three time points (i.e., 1 week [1W], 1 month [1M] and 3 months [3M]) after chemotherapy initiation. Representative day (and allowable range) for the three time points were defined as follows: 1W as Day 7 (Day 1 to 13), 1M as Day 28 (Day 14 to 55), and 3M as Day 84 (Day 56 to 111). If multiple values were present within a certain range, the value nearest to the representative day was used.

Supplementary Table S8 TSAT values after starting chemotherapy (grouped by tumor type)

	Time point	Baseline	1W	1M	3M
All	*n*	1,205	319	936	1,140
	TSAT (%)	***19.1*** [11.8–29.0]	21.8 [13.3–36.1]	21.8 [14.4–33.3]	20.9 [13.1–32.7]
n (%)	<20	640 (53.1)	144 (45.1)	411 (43.9)	530 (46.5)
	20≤ <50	520 (43.1)	126 (39.5)	415 (44.3)	497 (43.6)
	50≤	45 (3.7)	49 (15.4)	110 (11.8)	113 (9.9)
Stomach	*n*	208	47	115	151
	TSAT (%)	***17.6*** [10.4–26.3]	26.9 [14.4–38.6]	21.5 [13.3–35.3]	22.9 [13.7–34.1]
n (%)	<20	124 (59.6)	17 (36.2)	49 (42.6)	61 (40.4)
	20≤ <50	79 (38.0)	20 (42.5)	58 (50.4)	77 (51.0)
	50≤	5 (2.4)	10 (21.3)	8 (7.0)	13 (8.6)
Colorectum	*n*	166	28	112	129
	TSAT (%)	***18.1*** [10.5–26.0]	18.9 [9.9–26.4]	19.6 [11.8–30.4]	19.7 [12.2–30.4]
n (%)	<20	93 (56.0)	16 (57.1)	57 (50.9)	66 (51.2)
	20≤ <50	68 (41.0)	11 (39.3)	48 (42.9)	53 (41.1)
	50≤	5 (3.0)	1 (3.6)	7 (6.3)	10 (7.8)
Pancreas	*n*	203	32	144	181
	TSAT (%)	21.1 [14.3–29.7]	23.9 [13.4–46.4]	19.7 [13.4–27.7]	***17.1*** [11.5–25.7]
n (%)	<20	93 (45.8)	15 (46.9)	73 (50.7)	109 (60.2)
	20≤ <50	106 (52.2)	11 (34.4)	56 (38.9)	65 (35.9)
	50≤	4 (2.0)	6 (18.8)	15 (10.4)	7 (3.9)
Lung	*n*	130	80	179	243
	TSAT (%)	***13.8*** [10.4–22.9]	19.5 [12.6–39.2]	22.2 [13.7–34.2]	21.2 [14.0–34.6]
n (%)	<20	90 (69.2)	40 (50.0)	74 (41.3)	108 (44.4)
	20≤ <50	34 (26.1)	21 (26.3)	86 (48.0)	105 (43.2)
	50≤	6 (4.6)	19 (23.8)	19 (10.6)	30 (12.3)
Breast	*n*	87	13	73	102
	TSAT (%)	***19.1*** [12.4–27.1]	19.3 [12.8–35.3]	25.8 [19.1–33.2]	21.0 [12.8–29.6]
n (%)	<20	47 (54.0)	7 (53.9)	21 (28.8)	45 (44.1)
	20≤ <50	38 (43.7)	5 (38.5)	46 (63.0)	48 (47.1)
	50≤	2 (2.3)	1 (7.7)	6 (8.2)	9 (8.8)
Uterus	*n*	20	6	17	21
	TSAT (%)	***17.6*** [10.1–23.2]	23.3 [17.6–29.1]	26.2 [15.8–54.7]	26.6 [14.8–35.3]
n (%)	<20	11 (55.0)	3 (50.0)	6 (35.3)	7 (33.3)
	20≤ <50	9 (45.0)	3 (50.0)	5 (29.4)	11 (52.4)
	50≤	0 (0.0)	0 (0.0)	6 (35.3)	3 (14.3)
Ovary	*n*	27	3	21	14
	TSAT (%)	***14.1*** [8.7–19.4]	31.9 [24.8–35.5]	19.1 [15.6–29.7]	18.5 [16.1–26.0]
n (%)	<20	21 (77.8)	1 (33.3)	11 (52.4)	10 (71.4)
	20≤ <50	6 (22.2)	2 (66.7)	7 (33.3)	3 (21.4)
	50≤	0 (0.0)	0 (0.0)	3 (14.3)	1 (7.1)

Values are medians with IQRs, and the minimum values within each tumor type are indicated in boldface italics. These data were taken from patients who did not receive treatment for anemia (iron preparation, ESA, or RBC transfusion), radiotherapy, or surgery from Day -91 to 0. Laboratory values were continuously monitored at three time points (i.e., 1 week [1W], 1 month [1M] and 3 months [3M]) after chemotherapy initiation. Representative day (and allowable range) for the three time points were defined as follows: 1W as Day 7 (Day 1 to 13), 1M as Day 28 (Day 14 to 55), and 3M as Day 84 (Day 56 to 111). If multiple values were present within a certain range, the value nearest to the representative day was used.

Supplementary Table S9 Ferritin values after starting chemotherapy (grouped by tumor type)

	Time point	Baseline	1W	1M	3M
All	*n*	1,713	460	1,321	1,538
	Ferritin (ng/mL)	***154.0*** [59.6–333.0]	290.0 [103.2–606.7]	297.9 [112.0–595.3]	262.0 [98.8–549.5]
n (%)	<30	262 (15.3)	27 (5.9)	85 (6.4)	120 (7.8)
	30≤ <100	379 (22.1)	81 (17.6)	214 (16.2)	268 (17.4)
	100≤ ≤500	825 (48.2)	215 (46.7)	609 (46.1)	728 (47.3)
	500< ≤800	117 (6.8)	47 (10.2)	201 (15.2)	197 (12.8)
	800<	130 (7.6)	90 (19.6)	212 (16.1)	225 (14.6)
Stomach	*n*	297	62	169	194
	Ferritin (ng/mL)	***80.0*** [26.1–194.0]	166.3 [50.6–302.8]	146.1 [57.0–365.0]	155.7 [65.1–416.2]
n (%)	<30	85 (28.6)	8 (12.9)	21 (12.4)	21 (10.8)
	30≤ <100	83 (27.9)	15 (24.2)	48 (28.4)	46 (23.7)
	100≤ ≤500	111 (37.4)	30 (48.4)	76 (45.0)	91 (46.9)
	500< ≤800	9 (3.0)	3 (4.8)	7 (4.1)	12 (6.2)
	800<	9 (3.0)	6 (9.7)	17 (10.1)	24 (12.4)
Colorectum	*n*	199	31	119	132
	Ferritin (ng/mL)	***106.5*** [38.0–273.6]	229.0 [78.7–425.2]	145.6 [55.7–330.3]	113.6 [48.7–343.0]
n (%)	<30	41 (20.6)	2 (6.5)	16 (13.4)	18 (13.6)
	30≤ <100	55 (27.6)	8 (25.8)	34 (28.6)	44 (33.3)
	100≤ ≤500	85 (42.7)	16 (51.6)	57 (47.9)	49 (37.1)
	500< ≤800	12 (6.0)	2 (6.5)	5 (4.2)	8 (6.1)
	800<	6 (3.0)	3 (9.7)	7 (5.9)	13 (9.8)
Pancreas	*n*	213	47	160	202
	Ferritin (ng/mL)	***212.8*** [106.1–410.4]	411.7 [158.3–801.1]	352.9 [178.9–672.1]	294.6 [138.2–544.5]
n (%)	<30	16 (7.5)	1 (2.1)	5 (3.1)	8 (4.0)
	30≤ <100	31 (14.6)	6 (12.8)	10 (6.3)	29 (14.4)
	100≤ ≤500	131 (61.5)	21 (44.7)	86 (53.8)	111 (55.0)
	500< ≤800	15 (7.0)	7 (14.9)	29 (18.1)	25 (12.4)
	800<	20 (9.4)	12 (25.5)	30 (18.8)	29 (14.4)
Lung	*n*	278	129	292	370
	Ferritin (ng/mL)	***229.1*** [97.6–441.6]	340.1 [117.0–773.0]	396.7 [181.9–707.5]	380.7 [158.0–726.3]
n (%)	<30	23 (8.3)	5 (3.9)	8 (2.7)	15 (4.0)
	30≤ <100	49 (17.6)	18 (13.9)	37 (12.7)	46 (12.4)
	100≤ ≤500	144 (51.8)	60 (46.5)	131 (44.9)	166 (44.9)
	500< ≤800	26 (9.3)	15 (11.6)	60 (20.6)	62 (16.8)
	800<	36 (12.9)	31 (24.0)	56 (19.2)	81 (21.9)
Breast	*n*	84	16	91	121
	Ferritin (ng/mL)	***94.0*** [47.3–256.0]	207.4 [54.5–463.9]	243.0 [133.3–462.7]	219.7 [82.0–448.2]
n (%)	<30	14 (16.7)	1 (6.3)	5 (5.5)	8 (6.6)
	30≤ <100	31 (36.9)	5 (31.3)	14 (15.4)	27 (22.3)
	100≤ ≤500	29 (34.5)	7 (43.8)	50 (55.0)	60 (49.6)
	500< ≤800	2 (2.4)	2 (12.5)	12 (13.2)	15 (12.4)
	800<	8 (9.5)	1 (6.3)	10 (11.0)	11 (9.1)
Uterus	*n*	18	6	22	30
	Ferritin (ng/mL)	185.7 [74.3–347.8]	***161.4*** [111.9–414.9]	523.1 [204.3–776.0]	227.5 [108.0–388.2]
n (%)	<30	2 (11.1)	0 (0.0)	0 (0.0)	3 (10.0)
	30≤ <100	4 (22.2)	1 (16.7)	1 (4.5)	4 (13.3)
	100≤ ≤500	9 (50.0)	4 (66.7)	9 (40.9)	18 (60.0)
	500< ≤800	1 (5.6)	1 (16.7)	6 (27.3)	2 (6.7)
	800<	2 (11.1)	0 (0.0)	6 (27.3)	3 (10.0)
Ovary	*n*	33	7	15	24
	Ferritin (ng/mL)	190.0 [91.5–324.0]	283.0 [128.9–837.4]	***185.5*** [153.1–293.8]	294.9 [195.0–451.7]
n (%)	<30	3 (9.1)	0 (0.0)	1 (6.7)	0 (0.0)
	30≤ <100	6 (18.2)	2 (28.6)	1 (6.7)	2 (8.3)
	100≤ ≤500	23 (69.7)	2 (28.6)	11 (73.3)	16 (66.7)
	500< ≤800	1 (3.0)	1 (14.3)	2 (13.3)	3 (12.5)
	800<	0 (0.0)	2 (28.6)	0 (0.0)	3 (12.5)

Values are medians with IQRs, and the minimum values within each tumor type are indicated in boldface italics. These data were taken from patients who did not receive treatment for anemia (iron preparation, ESA, or RBC transfusion), radiotherapy, or surgery from Day -91 to 0. Laboratory values were continuously monitored at three time points (i.e., 1 week [1W], 1 month [1M] and 3 months [3M]) after chemotherapy initiation. Representative day (and allowable range) for the three time points were defined as follows: 1W as Day 7 (Day 1 to 13), 1M as Day 28 (Day 14 to 55), and 3M as Day 84 (Day 56 to 111). If multiple values were present within a certain range, the value nearest to the representative day was used.

Supplementary Table S10 Baseline demographics (eligible population of NCCN/ESMO classification)

	NCCN (*N* = 3,550)				ESMO (*N* = 1,683)			
	n	%	Median	IQR	*n*	%	Median	IQR
Age (year)			73	66–78			73	66–79
Female gender	1,351	38.1			693	41.2		
Hospitalization	1,996	56.2			936	55.6		
Solid tumor type*								
Stomach	892	25.1			446	26.5		
Colorectum	1,249	35.2			637	37.8		
Pancreas	302	8.5			146	8.7		
Lung	328	9.2			153	9.1		
Breast	149	4.2			55	3.3		
Uterus	72	2.0			39	2.3		
Ovary	67	1.9			37	2.2		
History of radiation therapy	199	5.6			89	5.3		
History of surgery	1,721	48.5			876	52.0		
Hb (g/dL)†	3,514	99.0	10.9	9.7–12.2	1,675	99.5	10.1	9.2–11.1
<8	180	5.1			65	3.9		
≥8 and <10	886	25.2			677	40.4		
≥10 and <LLN	1,781	50.7			842	50.3		
≥LLN	667	19.3			91	5.4		
MCV (fL)	3,501	98.6	89.2	84.3–93.7	1,668	99.1	88.2	82.9–93.1
<80	394	11.3			245	14.7		
≥80 and <100	2,900	82.8			1,317	79.0		
≥100	207	5.9			106	6.4		
Serum iron (μg/dL)	1,982	55.8	44.0	24.0–73.0	934	55.5	34.0	20.0–61.0
TIBC (μg/dL)	1,501	42.3	279.0	231.0–330.0	718	42.7	273.0	214.0–331.0
UIBC (μg/dL)	1,729	48.7	219.0	172.0–277.0	814	48.4	219.5	165.0–288.0
TSAT (%)**†	1,848	52.1	15.8	9.1–26.6	874	51.9	12.6	7.5–23.6
<20	1,135	61.4			599	68.5		
≥20 and <50	628	34.0			240	27.5		
≥50	85	4.6			35	4.0		
Serum ferritin (ng/mL)†	1,843	51.9	97.7	30.0–283.9	869	51.6	85.4	24.1–285.4
<30	460	25.0			251	28.9		
≥30 and <100	472	25.6			209	24.1		
≥100 and ≤500	704	38.2			298	34.3		
>500 and ≤800	98	5.3			53	6.1		
>800	109	5.9			58	6.7		
Serum vitamin B_{12} (pg/mL)	216	6.1	562.0	341.5–968.0	120	7.1	599.5	386.5–1112.0
≥200	203	94.0			114	95.0		
Serum folate (ng/mL)	189	5.3	7.4	4.9–10.8	106	6.3	7.5	5.1–12.4
≥3	170	89.9			96	90.6		
CRP (mg/dL)	3,367	94.8	0.3	0.1–1.8	1,613	95.8	0.4	0.1–2.0
≤0.5	1,919	57.0			875	54.2		
>0.5 and ≤1.0	325	9.7			163	10.1		
>1.0	1,123	33.4			575	35.6		

[*] The numbers of patients with solid tumors were counted during the month including Day 0 and the previous two months. Patients with multiple tumors were separately counted for each tumor type.

[**] TSAT was calculated using the data from the same day of either 'serum iron and TIBC' or 'serum iron and UIBC'.

[†] Hb, TSAT and ferritin were evaluated based on the period of Day -28 to 0, which is not identical to that used in Fig. 5.

Supplementary Table S11 Treatment of anemia before or after starting chemotherapy (grouped by tumor type)

**Stomach**	Before				After			
	n	%	Iron dose (mg)		*n*	%	Iron dose (mg)	
			Median	IQR			Median	IQR
Total	7,252	100			6,651	100		
Oral iron	1,216	16.8	2,800	1,300–5,375	1,121	16.9	6,700	2,835–8,900
SFO	554	7.6	360	160–640	208	3.1	300	160–640
FCM	34	0.5	500	500–1,000	8	0.1	500	500–1,000
FDI	0	0			1	<0.1	1,500	1,500–1,500
ESA	14	0.2			21	0.3		
RBC transfusion (Hb <10)	839	11.6			365	5.5		
**Colorectum**	Before				After			
	n	%	Iron dose (mg)		*n*	%	Iron dose (mg)	
			Median	IQR			Median	IQR
Total	12,576	100			12,062	100		
Oral iron	1,854	14.7	3,100	1,400–5,775	1,367	11.3	5,500	2,350–8,500
SFO	790	6.3	400	240–640	116	1.0	240	120–520
FCM	32	0.3	500	500–1,000	6	<0.1	500	500–1,000
FDI	0	0			0	0		
ESA	22	0.2			28	0.2		
RBC transfusion (Hb <10)	964	7.7			296	2.5		
**Pancreas**	Before				After			
	n	%	Iron dose (mg)		*n*	%	Iron dose (mg)	
			Median	IQR			Median	IQR
Total	5,034	100			4,396	100		
Oral iron	200	4.0	2,650	1,050–5,600	285	6.5	4,515	2,100–8,400
SFO	72	1.4	360	240–640	43	1.0	320	160–560
FCM	1	<0.1	500	500–500	10	0.2	500	500–1,000
FDI	0	0			0	0		
ESA	10	0.2			8	0.2		
RBC transfusion (Hb <10)	207	4.1			272	6.2		
**Lung**	Before				After			
	n	%	Iron dose (mg)		*n*	%	Iron dose (mg)	
			Median	IQR			Median	IQR
Total	12,039	100			10,772	100		
Oral iron	319	2.6	2,100	700–5,000	353	3.3	4,200	1,900–7,800
SFO	35	0.3	320	160–560	22	0.2	320	240–400
FCM	5	<0.1	1,000	500–1,000	2	<0.1	750	500–1,000
FDI	0	0			0	0		
ESA	14	0.1			17	0.2		
RBC transfusion (Hb <10)	150	1.2			333	3.1		
**Breast**	Before				After			
	n	%	Iron dose (mg)		*n*	%	Iron dose (mg)	
			Median	IQR			Median	IQR
Total	8,950	100			8,737	100		
Oral iron	306	3.4	2,250	900–4,800	511	5.8	2,900	1,500–6,100
SFO	53	0.6	240	120–560	26	0.3	220	80–360
FCM	4	<0.1	1,250	750–1,500	6	0.1	750	500–1,000
FDI	0	0			0	0		
ESA	8	0.1			10	0.1		
RBC transfusion (Hb <10)	84	0.9			122	1.4		
**Uterus**	Before				After			
	n	%	Iron dose (mg)		*n*	%	Iron dose (mg)	
			Median	IQR			Median	IQR
Total	2,417	100			2,333	100		
Oral iron	411	17.0	2,100	1,100–3,690	306	13.1	2,900	1.350–6,100
SFO	187	7.7	360	200–600	52	2.2	360	160–620
FCM	18	0.7	500	500–1,000	10	0.4	1,000	500–1,000
FDI	0	0			1	<0.1	1,500	1,500–1,500
ESA	14	0.6			1	<0.1		
RBC transfusion (Hb <10)	178	7.4			110	4.7		
**Ovary**	Before				After			
	n	%	Iron dose (mg)		*n*	%	Iron dose (mg)	
			Median	IQR			Median	IQR
Total	2,020	100			1,938	100		
Oral iron	212	10.5	1,600	717.5–3,000	213	11.0	2,400	900–5,100
SFO	151	7.5	360	240–480	28	1.4	620	200–840
FCM	12	0.6	500	500–1,000	7	0.4	500	500–500
FDI	3	0.1	1,000	500–1,500	3	0.2	1,000	1,000–2,000
ESA	2	0.1			1	<0.1		
RBC transfusion (Hb <10)	135	6.7			99	5.1		

Total shows all patients who have any observational period before starting chemotherapy (Day -84 to 0, shown as Before) or after starting chemotherapy (Day 1 to 84, shown as After), respectively. The iron dose represents the total cumulative iron dose during this period. Patients treated only with oral iron were grouped into 'Oral iron' group. Among patients treated with SFO, patients who took a combination of oral iron were grouped into the 'SFO' group. Among patients treated with FCM, patients who took a combination of oral iron and/or SFO were grouped into the 'FCM' group. Among patients treated with FDI, patients who took a combination of oral iron, SFO, and/or FCM were grouped into the 'FDI' group.

Supplementary Table S12 Effects of pretreatment with iron prior to chemotherapy on Hb values

		n	Iron dose (mg)	Hb (g/dL)		Pretreatment start	Other iron preparation combination			
							Oral iron		SFO	
				Pre	Post		*n*	Iron dose (mg)	*n*	Iron dose (mg)
Pretreatment started between Day -84 and -57	Oral iron group	173	4,200 (2,450–6,500)	9.8 (8.6–10.9)	11.1 (10.1–12.3)	Day -70.0 (-76.0, -63.0)	–	–	–	–
	SFO group	53	320 (160–800)	8.1 (7.2–9.2)	11.7 (10.3–12.7)	Day -69.0 (-80.0, -65.0)	30	4,500 (1,475–6,550)	–	–
	FCM group	2	500 (500–500)	5.3 (4.9–5.7)	11.3 (10.6–12.0)	Day -64.5 (-68.3, -60.8)	1	5,000 (5,000–5,000)	1	840 (840–840)
Pretreatment started between Day -56 and -29	Oral iron group	291	3,200 (1,600–4,700)	9.4 (8.5–10.7)	10.9 (9.7–12.0)	Day -42.0 (-48.5, -34.0)	–	–	–	–
	SFO group	130	400 (240–640)	8.5 (7.6–9.6)	11.2 (10.5–12.3)	Day -40.0 (-48.0, -34.0)	77	2,800 (1,500–4,300)	–	–
	FCM group	6	1,000 (1,000–1,375)	8.0 (7.7–9.1)	11.5 (11.3–12.4)	Day -47.5 (-53.0, -41.3)	2	3,000 (2,000–4,000)	1	80 (80–80)
Pretreatment started between Day -28 and -7	Oral iron group	447	1,400 (800–2,310)	9.4 (8.3–10.5)	10.0 (9.0–11.2)	Day -16.0 (-21.0, -11.0)	–	–	–	–
	SFO group	150	320 (210–590)	8.8 (7.9–9.7)	9.9 (8.8–11.1)	Day -17.5 (-22.0, -13.3)	69	1,400 (700–2,200)	–	–
	FCM group	14	750 (500–1,000)	8.7 (7.9–9.4)	9.7 (8.2–10.4)	Day -13.5 (-21.5, -9.8)	3	1,400 (1,350–1,700)	0	–

Eligible patients in this analysis have both ‘Pre’ and ‘Post’ Hb values. The data except for *n* are shown in medians ± IQRs. Patients treated only with oral iron were grouped into 'Oral iron' group. Among patients treated with SFO, patients who took a combination of oral iron were grouped into the 'SFO' group. Among patients treated with FCM, patients who took a combination of oral iron and/or SFO were grouped into the 'FCM' group. No patients received FDI.
